# Effect of COVID-19 on mortality due to diabetes mellitus in Brazil: A time series analysis from 2010 to 2023

**DOI:** 10.1371/journal.pone.0344419

**Published:** 2026-03-10

**Authors:** Larissa Otaviano Mesquita, Debora França dos Santos, Kenia Mara Baiocchi de Carvalho

**Affiliations:** 1 Graduate Program of Public Health, Faculty of Health Sciences, University of Brasilia, Brasilia, Brazil; Federal University of Ceara, BRAZIL

## Abstract

Diabetes mellitus (DM) is a major global public health concern, particularly in Brazil. This study aimed to analyze DM-related mortality trends before and during the pandemic to assess the possible influence of the COVID-19 pandemic on DM mortality in Brazil. Mortality was assessed using standardized mortality rates stratified by age group (20–29 years, 30–49 years, 50–69 years, 70 years or older), sex (male, female), and geographic region (North, Northeast, Southeast, South, and Midwest). Mortality rates were calculated using the Brazilian Mortality Information System and official national population estimates. An analytical ecological time series analysis was performed using the Prais–Winsten regression model to assess the DM-related mortality trends in two scenarios: before (2010–2019) and including the pandemic period (2010–2023). The annual percentage change (APC) and 95% confidence intervals (CIs) were calculated to synthesize the trends as decreasing, increasing, or stationary. DM-related mortality rates varied from 42.3 in 2010 to 35.9 per 100,000 deaths in 2023, with the highest values in the Northeastern region. An increase in mortality was observed from 2020 onwards. Regression analysis shows a decreasing trend in the pre-pandemic period (2010–2019) in women (−1.87%), 50–69 years (−1.24%), and ≥70 years (−1.21%). A decline was also observed in the Northeast (−1.06%), Southeast (−1.73%), and nationwide (−0.96%). However, after incorporating data from the pandemic period (2010–2023), the trend became stationary for individuals aged 50–69 years and ≥70 years, the Southeastern region, and Brazil. This change makes the expected mortality uncertain and highlights the need for effective coping strategies after the pandemic.

## Introduction

Diabetes mellitus (DM) is a chronic metabolic disorder that can lead to significant complications and impose high care demands and costs on public health systems [1–4]. It is one of the most prevalent noncommunicable chronic diseases (NCDs), and Brazil has the sixth highest number of adults living with the condition globally, with 15.7 million diagnosed individuals [4]. According to the Surveillance System of Risk and Protective Factors for Chronic Diseases by Telephone Survey (Portuguese acronym, Vigitel), in 2023, 10.2% of adults living in Brazilian state capitals reported a medical diagnosis of diabetes. The estimated prevalence in 2006 was 5.5% and showed a growing trend, reaching an annual increase of 0.26% by 2014. Similarly, DM-related mortality rates showed an increasing trend [4–7].

Morbidity and mortality associated with DM pose noticeable challenges to public health. It increasingly affects individuals in their productive years, leading to a decrease in the quality of life and premature death, as well as high costs for control and treatment [1,2,8,9]. Additionally, DM is often referred to as a “silent disease,” since only an estimated 50% of affected individuals receive a medical diagnosis. This lack of detection can contribute to increased disease severity and a higher risk of mortality. Moreover, population aging, especially the increase in combined lifestyle risk behaviors, makes future mortality estimates for this cause uncertain [4,10].

The coronavirus disease (COVID-19) pandemic has affected public health globally, with approximately 77.8 million people infected [11]. In Brazil, 37.5 million people have been infected, resulting in over 700,000 deaths [11]. Besides representing a major health issue by itself, causing excess mortality 1.2 times greater in 2020 and 2021 than that observed in Brazil in previous years, studies have highlighted the indirect impact of the COVID-19 pandemic [12]. Infection poses a disproportionate additional risk of causing more severe symptoms, complications, and death in individuals with chronic diseases, including DM [13,14]. Furthermore, the public health emergency triggered by COVID-19 has prompted the restructuring of healthcare services, resulting in a decrease in non-emergency medical appointments by the public health system and restricting preventive services essential for managing chronic conditions such as DM [15].

Before the pandemic outbreak, mortality rates from noncommunicable diseases in Brazil showed a consistently decreasing trend over the years, notably between 30 and 69 years of age, with DM presenting variations between regions. However, those analyses comprehended the four groups of NCDs and used adjusted estimates of mortality [16]. Studies analyzing the time trend of DM-related mortality using population-based data in Brazil date back to 2012 [17,18], indicating a significant time gap. Although it quantifies negative health aspects, the analysis of mortality rates offers crucial insights into public health and the effectiveness of the healthcare system, especially when directed toward preventable and premature outcomes. Time-series analysis is a relevant approach for revealing important patterns of change [19].

While previous research has addressed related aspects, this is the first study to evaluate DM mortality trends in Brazil using an appropriate time-series statistical method to estimate the effects of the COVID-19 pandemic on mortality patterns and geographic disparities. Therefore, this study aimed to evaluate the trend in DM- mortality before and during the pandemic period to assess the possible influence of the COVID-19 pandemic on the mortality trend of DM in Brazil.

## Materials and methods

### Study design and data source

**Fig 1:**
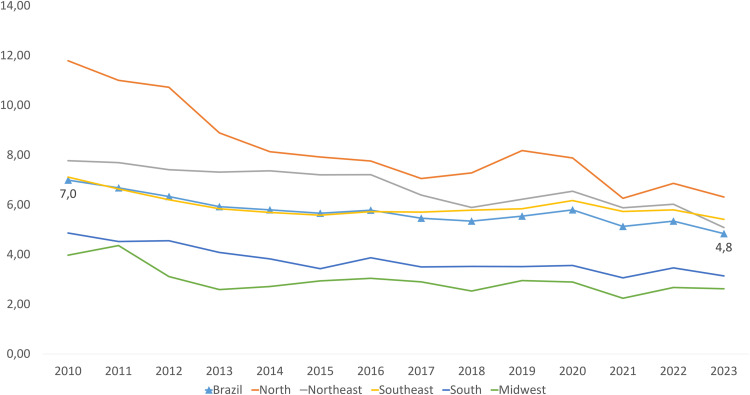
Proportion of deaths from ill-defined causes by region, 2010–2023. Mortality Information System. The proportion of ill-defined causes was calculated by dividing the number of deaths coded R00–R99 by the total number of deaths each year. Data are available in Supplementary Table 1 in S1 File.

This ecological study employs a time-series analysis using a quantitative approach. It was carried out using data on mortality caused by DM obtained from the Mortality Information System (Portuguese acronym, SIM) in Brazil, which is publicly available through the Brazilian Ministry of Health web page: https://datasus.saude.gov.br/informacoes-de-saude-tabnet/. Mortality data are highly reliable for indicator analysis, as the proportion of deaths from ill-defined causes—an indicator of data quality—has remained below 10% in all regions of Brazil since 2013 (Fig 1).

The population data used were estimated in 2024 by the Brazilian Institute of Geography and Statistics (Portuguese acronym: IBGE), based on the recent demographic census (2022), historical series of mortality, births, civil records, and other sources The data are publicly available at: https://www.ibge.gov.br/estatisticas/sociais/populacao/9109-projecao-da-populacao.html [20].

### Setting and quantitative variables

The unit of analysis was deaths by place of residence caused by DM, identified as deaths with the primary cause recorded under codes E10 to E14 in the 10th Revision of the International Statistical Classification of Diseases and Related Health Problems (ICD-10). These codes included all types of DM except gestational diabetes and were not stratified by type due to the high proportion of records registered as unspecified DM. Although this proportion has improved over time, it still accounted for 65% of all DM mortality records in 2023 (Supplementary Table 2 in S1 File) [18]. For descriptive analysis, mortality rates were calculated for individuals aged 20 years and older, based on annual observations from 2010 to 2023. The year 2023 was defined as the most recent year for which final mortality data were published, as established normatively [21]. These rates were calculated by dividing the total number of deaths in a population by the total population size, expressed per 100,000 inhabitants for a given year and geographic area, stratified by sex (male, female), Brazil’s regions (North, Northeast, Southeast, South, and Midwest), and age groups (20–29 years, 30–49 years, 50–69 years, and 70 years or more). The age groups were defined to provide a detailed understanding of premature mortality. Specifically, the range was segmented into a younger premature group (30–49 years) and an older premature group (50–69 years) to account for potential differences associated with the wide age variation within the premature mortality range. To undermine the effect of unequal age population composition in different geographic areas [22], mortality rates were standardized by age in 5-year brackets using the direct method, in which the population count from the 2010 Brazilian census served as the standard population. Descriptive analysis was conducted using control diagrams with pre-pandemic median and interquartile range values as standard limits. A total of 215 records with missing data for the analyzed variables were excluded, representing 0.3% of all DM-related death records during the study period.

### Trend analysis

The Prais–Winsten regression method was employed to analyze mortality rate trends. The model is described by the expression: Y_t_ = β_0_ + β_1t_ + e_t_, in which β_0_ is the constant term (intercept), 𝛽_1_ is the slope of the line (regression coefficient or scale factor), and e_t_, is the random error. Trends were estimated on a logarithmic scale over two different time frames: 2010–2019 (before the pandemic) and 2010–2023 (including the pandemic period), to identify potential variations associated with the COVID-19 pandemic. Since the model used log-transformed mortality rates, the annual percentage change (APC) was directly derived from the estimated regression coefficient (β₁) using the formula [23,24]:


$$APC\;=\;[-\text{1}+{\text{1}\text{0}}^{\beta \text{1}}*\text{1}\text{0}\text{0}\%$$

The APC is a trend measure that synthesizes variations using weighted averages over a predetermined period. The 95% confidence intervals for the APC were calculated, and the trend was considered increasing if both the APC and its confidence intervals had a positive outcome; if negative, the trend was considered decreasing, and it was considered stationary when there was no statistical significance (p > 0.05). Autocorrelation correction and residual independence were evaluated using the Durbin–Watson statistic, and the majority of the results were approximately two.

The database was extracted using TabWin version 4.15. Data processing, descriptive analysis, and estimation were performed using the statistical software R, version 4.4.0.

### Ethics statement

The study design was in accordance with the National Board of Health Resolution No. 510/2016, based on the principles expressed in the Declaration of Helsinki. This study did not require ethical approval from competent authorities, as it was conducted using secondary data from Brazilian public domain databases, with no nominal identification of subjects.

## Results

Between 2010 and 2023, DM caused approximately 900,000 deaths in Brazil, resulting in an average mortality rate of 40.3 deaths per 100,000 inhabitants annually, which varied from 42.3 in 2010 to 35.9 in 2023. In the pre-pandemic period, mortality was 38.1 in 2019. DM-related mortality was similar for both men and women in 2010 (42.1 and 42.0 deaths per 100,000, respectively), when it began to decline in women but remained steady in men, creating divergent trends that resulted in rates of 38.4 for women and 33.7 for men by 2023. The year 2020 showed an increase in diabetes mortality for both sexes but remained more expressive in men in comparison with women every year between 2010 and 2023 (Table 1).

**Table 1:** Standardized diabetes mellitus mortality rates, by sex, age group, and Brazilian regions, 2010–2023.

Analysis unit	2010	2011	2012	2013	2014	2015	2016	2017	2018	2019	2020	2021	2022	2023
**Sex**														
Men	42.1	43.6	41.2	41.1	39.9	40.1	40.2	40.8	40.8	40.4	45.7	45.4	42.8	38.4
Women	42.0	42.5	40.5	39.7	38.1	37.7	37.4	36.9	36.3	36.0	38.9	39.6	37.3	33.7
**Age group**														
20–29 years	1.1	1.1	1.1	1.1	1.2	1.1	1.3	1.3	1.3	1.3	1.8	1.6	1.5	1.3
30–49 years	6.2	6.5	6.1	6.1	5.9	6.0	6.2	6.0	6.3	6.2	7.7	7.7	6.7	6.6
50–69 years	64.3	65.3	61.9	60.3	58.4	58.4	58.6	58.7	58.9	57.5	63.8	63.5	58.6	53.0
≥ 70 years	337.4	345.4	329.3	329.3	318.1	316.4	313.1	314.1	308.7	309.0	334.3	339.6	327.5	291.9
**Region**														
North	43.5	48.7	47.1	46.9	48.2	49.1	47.6	53.6	55.6	49.8	57.0	49.2	44.9	42.3
Northeast	50.2	54.6	51.2	50.4	49.6	50.7	49.7	51.0	48.6	45.5	53.7	50.1	48.9	41.9
Southeast	38.0	37.3	35.0	34.1	32.7	31.9	31.9	32.6	34.2	32.3	37.2	37.5	34.0	31.5
South	41.5	40.3	39.5	41.8	37.2	36.6	38.2	38.9	43.9	39.5	41.3	43.7	43.1	38.2
Midwest	40.2	39.6	39.4	36.9	38.9	38.5	37.6	41.2	41.6	36.5	38.4	36.0	34.4	32.9
Brazil	42.3	43.2	41.0	40.5	39.1	39.0	38.8	40.1	41.1	38.1	43.4	42.3	39.9	35.9

Mortality Information System; Mortality rates are derived from the ratio of deaths by population, multiplied by 100,000, and standardized by age. Deaths per 100,000 inhabitants.

**Fig 2:**
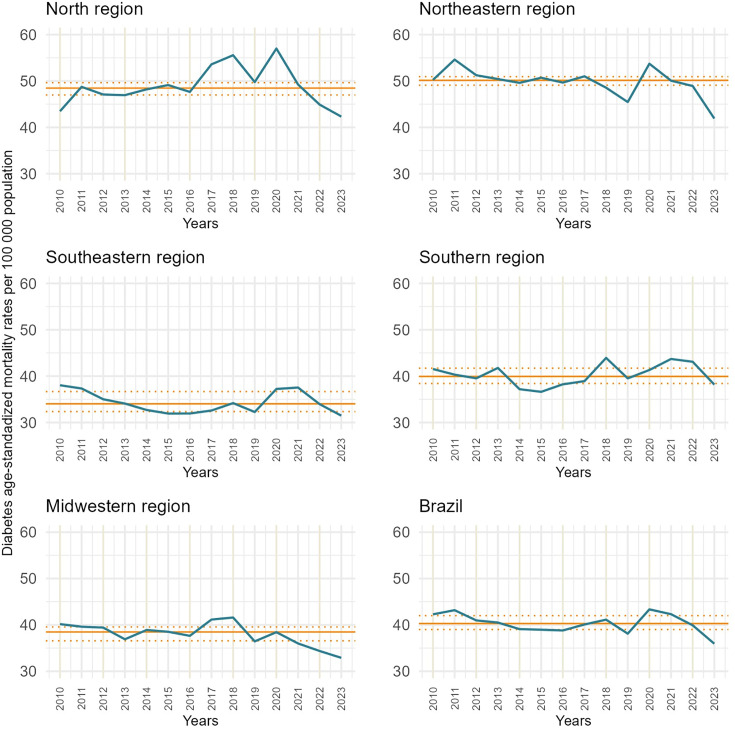
Diabetes mortality rates median value and interquartile range per sex, age group, and region, 2010–2023. Mortality Information System. Median and interquartile ranges were based on mortality rates before the pandemic years (2010–2019); mortality rates were derived from the division of deaths by populations, multiplied by 100,000, age-standardized. Represents the deaths per 100,000 inhabitants. Blue line: DM mortality | Solid orange line: pre-pandemic median | Dashed orange lines: interquartile range.

Descriptive data showed an increase in mortality with age. Geographic disparities were observed, with higher mortality rates in the north and northeast regions compared to other regions. The Northern region reached the highest mortality rate among all regions (57.0 deaths/100,000 inhabitants in 2020) (Table 1). Fig 2 shows the fluctuations in mortality in each region between 2010 and 2023. All regions, except for the Midwest, showed variations in DM-related mortality during the pandemic years, exceeding the interquartile range control limits, indicating a deviation from the historical pattern of the indicator’s behavior.

The trend analysis results showed that diabetes mortality in Brazil decreased by 0.96% per year before the pandemic. However, during the pandemic years, mortality rates remained stable. Considering sex, men maintained a stable mortality rate, while the trend in women continued decreasing with the inclusion of data after the pandemic, with a reduction in the APC from 1.87% to 1.33% per year. Conversely, age-group analysis revealed a consistent upward trend in mortality among young adults and a stationary trend in the 30–49-year-old age group during both periods. The pre-pandemic mortality trend for people aged ≥50 years showed a decline, which stabilized with the inclusion of the pandemic data (Table 2).

**Table 2:** Prais–Winsten regression model before (2010–2019) and including pandemic period (2010–2023), by sex, age group and region of residence.

Analysis unit	Before pandemic period (2010–2019)			Including pandemic period (2010–2023)		
	APC (%)	95% CI*	Trend	APC (%)	95% CI*	Trend
**Sex**						
Men	−0.55	(−1.17; 0.08)	Stationary	−0.15	(−1.22; 0.92)	Stationary
Women	−1.87	(−2.32; −1.42)	Decreasing	−1.33	(−2.28; −0.37)	Decreasing
**Age group**						
20–29 years	2.45	(1.45; 3.45)	Increasing	2.79	(0.86; 4.77)	Increasing
30–49 years	−0.13	(−0.88; 0.63)	Stationary	0.96	(−0.45; 2.39)	Stationary
50–69 years	−1.24	(−2.03; −0.44)	Decreasing	−0.96	(−2.04; 0.12)	Stationary
≥ 70 years	−1.21	(−1.52; −0.89)	Decreasing	−0.68	(−1.55; 0.20)	Stationary
**Region**						
North	1.78	(0.68; 2.71)	Increasing	0.13	(−1.65; 1.94)	Stationary
Northeast	−1.06	(−1.73; −0.38)	Decreasing	−0.79	(−1.44; −0.14)	Decreasing
Southeast	−1.73	(−3.19; −0.23)	Decreasing	−0.86	(−2.32; 0.62)	Stationary
South	−0.17	(−1.79; 1.49)	Stationary	0.22	(−0.75; 1.20)	Stationary
Midwest	−0.09	(−1.14; 0.97)	Stationary	−1.13	(−2.13; −0.12)	Decreasing
**Brazil**	**−0.96**	**(−1.86; −0.05)**	**Decreasing**	**−0.58**	**(−1.46; 0.31)**	**Stationary**

Mortality Information System; APC: annual percentage change; 95% CI*: 95% confidence intervals.

The trends for the three regions (North, Southeast, and Midwest) shifted when comparing the time series analysis before and including the pandemic period scenarios (Table 2). The inclusion of pandemic-period mortality data revealed that most geographic areas analyzed presented an uncertain trend, leading to a stagnant outcome. The trend changed from declining to stationary in the Southeastern region and in Brazil, indicating that the continuous decreasing trend attained during the pre-pandemic period was lost. The Northeast and South were the only two regions that maintained their original trend direction. The Northeast showed a consistent annual decrease, with higher mortality during the pandemic period, as indicated in the control diagram, but not sufficient to change the direction of the trend.

## Discussion

This is the first study to analyze the influence of the COVID-19 pandemic on DM mortality trends in Brazil [25]. It enriches the analysis of DM-related mortality trends, which are poorly characterized in Latin American countries, with more studies being conducted in high-income countries in North America, Europe, and the Asia-Pacific region, showing a markedly declining trend in DM-related mortality [17]. Notably, we applied a robust time-series analysis technique to epidemiology and public health surveillance to improve our knowledge of chronic disease behavior during health outbreak emergencies.

DM mortality rates in Brazil generally declined until 2019. However, this reduction was geographically uneven, indicating significant differences in both trend and descriptive mortality rate analyses across regions. Lower-income and lower-education regions, such as the northern and northeastern regions of Brazil, are consistently associated with a higher risk of DM-related death [26,27]. Low-wage status is associated with longer hospital stays for DM [28] and a higher frequency of DM-related complications [29]. In contrast, individuals with higher levels of education are more likely to receive DM management self-care medical advice and to complete all recommended preventive follow-up tests compared to those with less education [30]. Similar results were found in the Americas region, where mortality due to diabetes tends to increase in low- and middle-income countries, while it has declined in high-income countries, such as those in North America [17,31].

The quality of available primary healthcare is another factor associated with higher mortality rates. DM is considered a preventable cause of death in Brazil when access to affordable healthcare services is adequate [9]. Primary care services are pivotal in managing and preventing mortality caused by DM. Having a regular source of these primary care services is associated with a decreased risk of hospitalization, and effective symptom management is associated with fewer hospital admissions owing to short-term DM complications. However, the quality of care depends on good access, the availability of health practitioners, and resources [28], which also have significant geographic disparities related to socioeconomic status [32], particularly evident in the North and parts of the Northeast, which face greater challenges in accessing healthcare services [33]. The Southern and Southeastern regions consistently report higher rates of medical consultations and better infrastructure, contributing to the decreasing trend in diabetes mortality despite having an older population [34].

During the COVID-19 pandemic period, the health crisis compromised the standards of care for individuals with DM despite the country’s well-established healthcare system and strategies applied to protect and guarantee access to health services in Brazil [35]. The pandemic scenario imposed a reorganization of health services, which reduced elective appointments for chronically ill patients and a higher probability of healthcare cancellations, especially in the northern region [36,37]. These changes caused reduced glycemic control in patients with DM and an increase in relevant risk factors for DM complications, such as unhealthy food intake and physical inactivity [38]. Consequently, DM-related adverse outcomes were worse than those in the pre-pandemic period, including all-cause and DM-related mortality, amputations, sight loss, and a higher probability of death [25].

DM is also a well-documented risk factor for adverse COVID-19 outcomes, such as severe illness, ICU admission, and an amplified risk of mortality of 1.75 when compared with non-diabetic patients [39]. This association occurs because SARS-CoV-2 triggers an inflammatory response that causes insulin resistance and damages insulin-producing cells. Patients with DM are at higher risk of developing severe infections and serious complications [40]. The combination of these direct physiological effects and subsequent indirect effects likely explains the changes observed in this study and highlights key areas for targeted interventions.

DM is one of the most common causes of death, and according to the current study, it is more common in men than in women. Although the estimation of sex disparities in DM mortality has not received much attention, studies have found similar results for DM mortality and noncommunicable diseases [41]. Men with DM at 40 years of age are expected to lose more years of life than women, although the risk of developing DM is higher among women [42,43]. Historically, DM-related mortality has been predominant in women since 1980 and shifted to men in 2012 owing to a mean annual increase of 3.4% between 1980 and 2005 [44]. Environmental and physiological factors such as testosterone deficiency, a higher prevalence of smoking, obesity, and high blood pressure can lead to a higher risk of mortality [45,46]. Additionally, men tend to seek healthcare services less frequently and have a higher prevalence of risk factors for noncommunicable diseases [34]. Similar trends are observed in countries in the American region, where mortality rates have increased faster in men than in women [31].

Considering age, the trend shifted to stationary at ≥70 years of age including pandemic data, which is expected due to age being an important risk factor for DM diagnoses and offering an additional risk for complications of COVID-19. However, older women were 82% more likely to have healthcare canceled due to the pandemic and 76% less likely to seek care for COVID-19 symptoms, which can be attributed to concern about contracting the infection in healthcare facilities [37]. The present study identified an increase in mortality among young adults aged 20–29 years, potentially linked to the rise in DM risk factors, such as inadequate food intake and reduced access to healthcare services during the pandemic, although further investigation is necessary to confirm and better understand these results [47,48]. A marked disparity was observed across age groups: younger adults (20–29 years) consistently exhibited an upward mortality trend in all scenarios, whereas mortality declined among individuals in the older premature-age group (50–69 years) as well as among those aged 70 years and over. Increases in diabetes mortality among younger adults have also been observed in Latin American countries [49].

Elevated mortality associated with COVID-19 can be anticipated due to higher exposure to infection, compounded by a greater prevalence of comorbidities and reduced access to adequate healthcare. Understanding disease mortality has become a leading strategy for significantly reducing deaths, particularly among the most vulnerable segments of the population, who are disproportionately affected in Brazil [28]. Given the high prevalence of DM, it is fundamental to fully comprehend the traits, vulnerability, and effects of COVID-19 on patients with DM to improve standards of care for these patients in future emergency situations.

Standardized mortality rates were the only metric used in this study because they smooth the influence of population age composition on crude death rates, permitting a better comparison between two or more population groups with different age structures [22]. The rates, measured annually, are widely used in monitoring NCD indicators and are consistent with the temporality of the databases employed in this analysis. Further evaluation using a different time scale may reveal sub-annual variations. Although mortality data evaluation constitutes passive surveillance, in which there is no possibility of changing the outcome, it remains reliable information because death certificates are mandatory in American countries and reflect the severity of diseases as public health issues. However, mortality data accuracy can be influenced by medical diagnostics, errors in classifying causes of death, and the presence of competing risks, which is the reduced probability of certain causes of death being noted because of the age and sex structure of the population. Notably, the emergence of the pandemic may have affected the quality of death reporting, including under-registration or suppression of diabetes as the main cause of death in patients who died with COVID-19 infection, thereby influencing trend estimates [22].

We emphasize that the statistically significant increasing or decreasing trends reflect the abrupt rise in DM mortality during the pandemic period when considering the entire analysis timeframe. Since Prais-Winsten regression estimates a linear trend over the full period, the exceptionally high values observed during the pandemic reduce the overall slope of the fitted line. Therefore, the appropriate interpretation is not a reversed underlying trend, but rather an apparent stabilization caused by disruptions related to the pandemic years, particularly in series that exhibited a pre-pandemic decline. [22] Nonetheless, examining shifts in trend trajectories due to the COVID-19 pandemic remains a highly relevant approach for understanding the dynamics of emergency health events on chronic disease patterns. Further research is required to identify which pandemic-related factors are most closely associated with the sudden shift in mortality data behavior and the time required for past patterns to reappear.

## Conclusion

There was a declining trend in DM-related mortality estimates during the pre-pandemic period among individuals aged 50 years and older, in the Southeastern region, as well as in Brazil overall. However, after incorporating data from the pandemic period (2010–2023), these trends stabilized, creating uncertainty about future DM-related mortality projections. This finding highlights the need for effective post-pandemic strategies to mitigate potential adverse outcomes associated with urgent emergency health situations.

## Supporting information

S1 File The supplementary tables described are publicly available in the repository: https://doi.org/10.6084/m9.figshare.31281523. (DOCX)
