# Supplementary material for: Effect of COVID-19 on mortality due to diabetes mellitus in Brazil: A time series analysis from 2010 to 2023
Source: PLoS One. 2026 Mar 10;21(3):e0344419. doi: 10.1371/journal.pone.0344419 (PMC12974813; doi:10.1371/journal.pone.0344419)
Supplement: S1 File — (DOCX) [file pone.0344419.s001.docx]

Suppl. Table 1. Proportion of deaths from ill-defined causes, 2010-2023

Mortality Information System. The proportion of ill-defined causes was calculated by dividing the number of deaths coded R00–R99 by the total number of deaths each year.

Suppl. Table 2. Proportion of unspecified Diabetes Mellitus cause of death, 2010-2023

Mortality Information System. The proportion of unspecified diabetes mellitus as the cause of death was calculated by dividing the number of deaths coded with specific diabetes ICD-10 by the total number of diabetes deaths in each year.

Suppl. Table 3. Number of deaths caused by DM in Brazilian regions, 2010-2023

Mortality Information System. Number of diabetes deaths coded under E10–E14.

Suppl. Table 4. Population in Brazilian regions, 2010-2023.

Brazilian Institute of Geography and Statistics. Population estimates, 2010-2023

The supplementary tables described are publicly available in the repository: https://doi.org/10.6084/m9.figshare.31281523
